# Effect of molecular hydrogen, a novelly-established antioxidant, on the retinal degeneration of hereditary retinitis pigmentosa: an *in-vivo* study

**DOI:** 10.3389/fphar.2023.1294315

**Published:** 2024-04-03

**Authors:** Weiming Yan, Qiurui He, Pan Long, Tao Chen, Lei Zhang, Haiyan Wang

**Affiliations:** ^1^ The Shaanxi Eye Hospital, Xi’an People’s Hospital, Xi’an Fourth Hospital, Xi’an, China; ^2^ The Third Hospital of Zhangzhou, Zhangzhou, China; ^3^ Fuzong Clinical Medical College of Fujian Medical University, Dongfang Hospital Affiliated to Xiamen University, Fuzhou, China; ^4^ The General Hospital of Western Theatre Command, PLA, Chengdu, China; ^5^ Center of Clinical Aerospace Medicine, Air Force Military Medical University, Xi’an, China

**Keywords:** retinitis pigmentosa, molecular hydrogen, retina, photoreceptors, antioxidant

## Abstract

**Objective** Our research was performed in order to explore the effects of molecular hydrogen (H_2_), a novelly-established antioxidant, on the retinal degeneration in *rd1* mice, an animal model of inherited retinitis pigmentosa (RP).

**Methods** The *rd1* mice were divided randomly into control and H_2_ intervention groups. Mice from other groups received H_2_ intervention in three modes, two modes of the hydrogen gas (HG) and one model of hydrogen-rich saline (HRS). At 14 days post born (P14) and P21, various indicators were detected in all mice, including eletroretinogram (ERG), fundus phography, optical coherence tomography (OCT), and retinal immunotaining of microglia cells’ marker, Iba1.

**Results** The ERG amplitude in mice from the control and H_2_ intervention groups showed no statistical differences (*p >* 0.05). At P14 and P21, no significant difference in the distance from the retinal pigment epithelium to the outer plexiform layer on OCT from mice of the above two groups was found (*p >* 0.05). The thickness of the outer nuclear layer (ONL) in mice at P14 and P21 showed no statistical differences between the control group and the H_2_ intervention group (*p >* 0.05). In the aspect of the number of Iba1-positive cells, we did not found any significant differences between the two groups (*p >* 0.05).

**Conclusion** Different forms of H_2_ intervention (hydrogen-rich saline and hydrogen gas) had no obvious effects on the course of retinal degeneration in *rd1* mice. The specific mechanism of photoreceptor degeneration in the hereditary RP mouse model may be different, requiring different medical interventions.

## Introduction

Retinitis pigmentosa (RP) has been well-known as a group of hereditary retinal diseases, which reserve the characteristics of progressive photoreceptor apoptosis and retinal degeneration (Liu et al., 2022). RP, with a worldwide prevalence of about 1/4,000, could lead to severe vision loss and blindness, causing great suffering for the life and family of patients (Daiger et al., 2013). Although gene therapy shows great potential in the treatment of RP, the high heterogeneity of more than 2000 mutation points makes the therapeutic effect far from satisfactory (Dvoriantchikova et al., 2022). Photoreceptor apoptosis is a common process in RP, and is the main cause of visual impairment. However, no effective medications currently exist to combat this in the clinic. New medicines for delaying or ameliorating RP are urgently required. Oxidative stress has been argued to be a vital part of evoking and enlarging the photoreceptor apoptosis in RP (Kanan et al., 2022). Data from previous researches suggested that substances that reduce oxidative stress were able to alleviate retinal degeneration in an RP mice model (Vingolo et al., 2022).

Recently, molecular hydrogen (H_2_) has emerged as a novel antioxidant due to its inherent properties of anti-oxidation and anti-inflammation (Tian et al., 2021). With its relatively low molecular mass, it can rapidly disseminate and penetrate cell membranes, enabling a broad spectrum of biological effects (Nogueira and Branco, 2021). H_2_ has been documented to exhibit therapeutic effects in a multitude of diseases, primarily by selectively diminishing the amount of reactive oxygen species (ROS) (Zhang et al., 2022a). In addition, its application of hydrogen gas (HG) has recently been recommended in the Coronavirus disease-2019 (COVID-19) pneumonia treatment guidelines of China recently (Alwazeer et al., 2021). In our previous investigation, it was observed that hydrogen-rich saline (HRS) effectively suppressed the progression of retinal degeneration in a rat model of induced RP (Chen et al., 2016; Yan et al., 2017a). However, the impact of H_2_ on retinal degeneration in inherited RP remains unexplored in existing literature.

In the current study, different forms of H_2_ were administered to explore their effect on retinal degeneration in *rd1* mice, a commonly employed inherited mouse model that exhibits key pathological traits of human RP (Nishiguchi et al., 2015; Leclercq et al., 2021). Our goal was to observe whether H_2_ intervention could alleviate photoreceptor degeneration in this inherited RP model, in order to expand the application scope of H_2_ and provide new intervention ideas for the treatment of RP.

## Materials and methods

### Animal preparation

The *rd1* mice used in this study were provided by the Aerospace Clinical Medicine Department of the Air Force Medical University (License no. #SYXK 2012-004) (Yan W. et al., 2017). The mice were housed under controlled environmental conditions, with a room temperature of 22°C–25°C and a standard light-dark cycle of 12 h of darkness followed by 12 h of light. All animal handling followed the Association for Research in Vision and Ophthalmology (ARVO) Statement and was approved by the Animal Care and Use Committee of the Fuzong Clinical Medical College of Fujian Medical University.

### H_2_ administration

#### For HRS

Briefly, HRS was prepared by dissolving hydrogen gas in saline under high pressure (0.4 MPa) for 6 h, resulting in a supersaturated solution. Fresh HRS was prepared on a weekly basis to ensure a concentration above 0.6 mM, which was verified using a gas chromatographic method as described previously (Yan et al., 2017c). The HRS was stored in an aluminum bag without any dead volume at a temperature of 4°C, under normal atmospheric pressure.

The *rd1* mice were randomly divided into the control and HRS groups (n = 6). Mice in the HRS group were administered a daily intraperitoneal injection of 10 mL/kg HRS from the 7 days post born (P7) until P21. Mice in the control group received daily intraperitoneal injections of the same dose of saline. The corresponding indicators of the HRS-induced effects were observed at P14 and P21.

#### For HG

As previously reported (Yan et al., 2018), to generate a high concentration of H_2_, an AMS-H-01 hydrogen oxygen nebulizer (Asclepius, Shanghai, China) was utilized, capable of extracting 67% H_2_ and 33% O_2_ from water. The H_2_-O_2_ mixture was then diluted with N_2_ (nitrogen gas, product code #12321128, Shaanxi XingHua Chemical Co. LTD., Xi’an, China) to achieve a standard O_2_ concentration of 21%. As a result, a final H_2_ concentration of 44% was attained.

**FIGURE 1:**
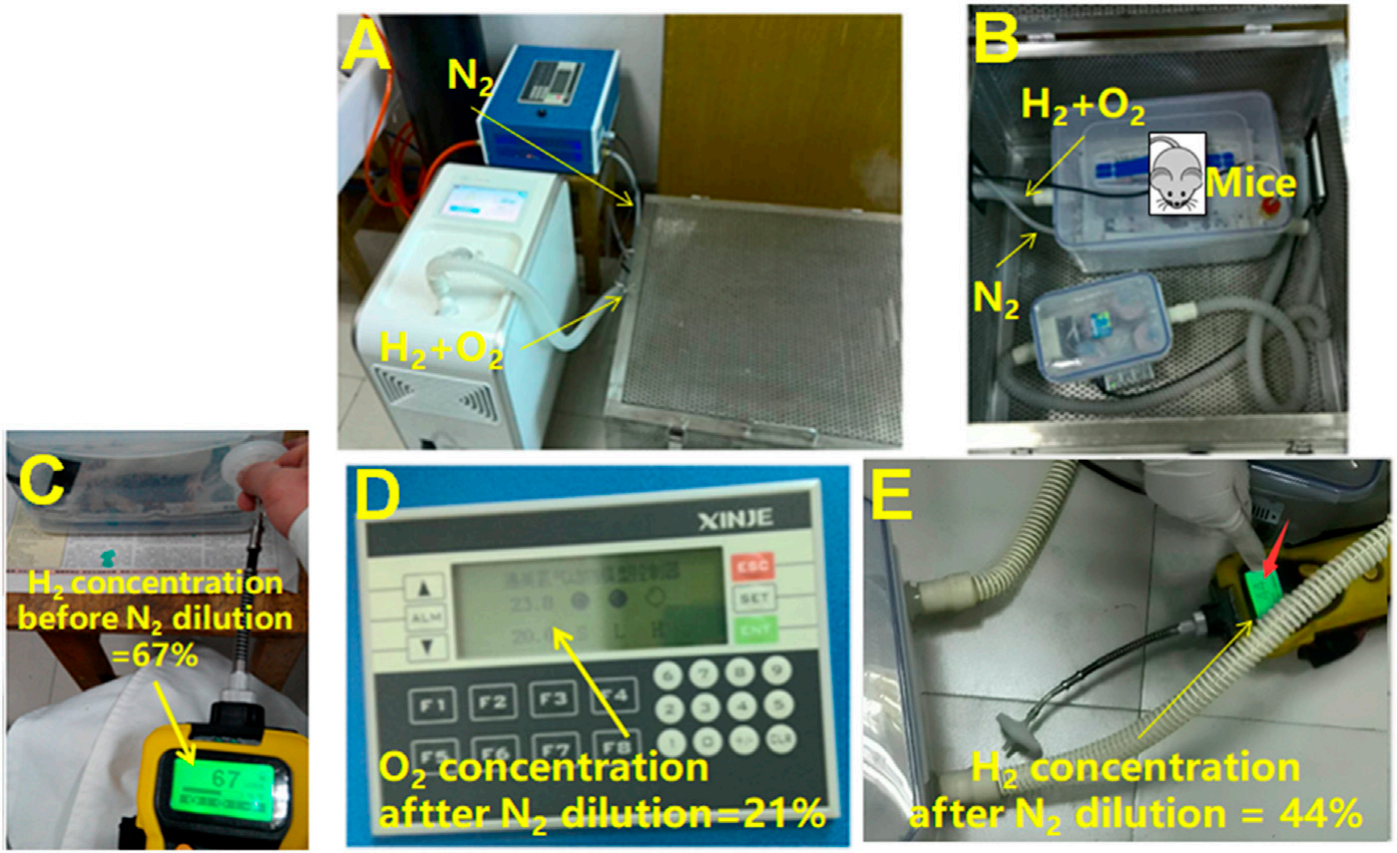
Schematic of the experimental process for studying the effects of hydrogen gas on RP on *rd1* mice. **(A)** Overview of the entire experiment, including hydrogen and oxygen produced by water electrolysis and nitrogen extracted from a nitrogen cylinder, which were introduced into the cage containing the mice via the corresponding pipelines. **(B)** The introduction of gases to the mouse cage, with the thin tube being the nitrogen inlet and the thick white tube being the hydrogen-oxygen mixed gas inlet. **(C)** Detection of hydrogen gas concentration in the cage using a hydrogen gas analyzer. Before the use of nitrogen for dilution, the hydrogen concentration was 67%, as shown on the detector screen. **(D)** Detection of oxygen gas concentration inside the cage using an oxygen gas analyzer. Dilution of the gases in the cage with nitrogen resulted in an oxygen concentration of 21%. **(E)** Detection of hydrogen gas concentration inside the cage again using a hydrogen gas analyzer. With the use of nitrogen for dilution, resulting in an oxygen concentration of 21%, the hydrogen concentration was 44%.

The *rd1* mice were randomly divided into three groups: control, hydrogen 1 group (HG1), and hydrogen 2 group (HG2) (n = 6). In the HG1 group, the mice inhaled a high concentration of hydrogen gas (44%) for 4 h once a day in a sealed container from birth to P21. The HG2 group mice, on the other hand, inhaled the same high concentration of hydrogen gas (44%) continuously for 24 h each day from birth to P21. The gas flow rate was maintained at 3 L/min. Throughout the inhalation process, the mice were awake and allowed to move freely within a designated gas chamber. The concentration of H_2_ in the chamber was monitored using thermal trace GC ultra-gas chromatography (Thermo Fisher, MA, USA). Mice in the control group were raised in a container from birth to P21 with normal environmental gas. The corresponding indicators were observed at P14 and P21 (Figure 1, Supplementary Figure S1 and Supplementary Table S1).

### ERG examination

Electroretinography (ERG) was conducted following the methods described in a previous study (Yan W. et al., 2017). All ERG parameters were recorded and analyzed in accordance with the guidelines provided by the International Society for Clinical Electrophysiology of Vision (ISCEV), by a technician who was unaware of the animal grouping and treatment (McCulloch et al., 2015). Briefly, mice underwent dark adaptation for more than 12 h before the experiment and were then deeply anesthetized. Pupils were dilated by applying a 0.5% of tropicamide ophthalmic solution (Shenyang XingjiCorporation, China), and a ring electrode was attached to the center of the cornea as the active electrode. Reference and ground electrodes were inserted beneath the skin and tail, respectively. The dark-adapted 3.0 ERG responses were recorded using a full-field (Ganzfeld) stimulation and a computer system (RETI port; Roland, Germany) with a light intensity of 3.0 cd^.^s^.^m^−2^ under scotopic conditions. All operations were conducted under scotopic conditions, with the help of a dim red light to maximize retinal sensitivity according to the guidelines of ISCEV.

### Fundus photography and OCT scanning

The animals were administered a combination of pentobarbital (Sigma-Aldrich, US) and Sumianxin II (Dunhua, China) via injection for anesthesia, and their pupils were dilated. Sodium hyaluronate gel (Jinan, China) was applied to cover the corneas, which were then attached to the camera lens of a Retinal Imaging System and 4D-ISOCT Microscope Imaging System (ISOCT, OptoProbe, Canada) to capture fundus and optical coherence tomography (OCT) images (Yan et al., 2021). To measure the distance between the inner border of the outer plexiform layer (OPL) and the retinal pigment epithelium (RPE), 400 μm from both sides of the optic disc, the OCT Image Analysis Software was utilized.

### Measurement of outer nuclear layer (ONL) thickness on the retina histology

At P14 and P21, mice (n = 6) were euthanized with an intraperitoneal injection of a lethal dose of sodium pentobarbital, and their eyes were quickly removed. The eyecups were collected and embedded in paraffin. Serial sections with a thickness of 3 μm were sliced. Three sections from each eye, including the optic nerve, were stained with hematoxylin and eosin (HE). Images of the sections were captured using a digital imaging system (DP71, Olympus, Japan) and analyzed by counting the number of rows of the outer nuclear layer (ONL) at high magnification (×400) in a region located 200 μm away from the optic nerve on both sides.

### Immunofluorescence staining of Iba1

Vertical retinal sections were deparaffinized in xylene and then rehydrated using a series of graded ethanol solutions. For antigen retrieval, the sections underwent heat treatment in a microwave and were subsequently incubated with 10% normal goat serum (Boster, Wuhan, Hubei Province, China) for 1 h at room temperature. Immunostaining was performed by applying a polyclonal rabbit anti-ionized calcium-binding adapter molecule 1 (Iba1, Wako Chemicals, Japan) antibody at a 1:500 dilution to the slides, which were then left overnight at 4°C. After rinsing with PBS, a secondary antibody (goat anti-rabbit IgG conjugated to Alexa Fluor 594, #ZF-0516, ZSGB-BIO, Beijing, China) was applied at a 1:500 dilution. DAPI (Beyotime, Nantong, China) was used for counterstaining the retinal sections. Images of retinas located 200 μm temporal to the optic nerve were captured using a digital immunofluorescence microscope imaging system (DP71, Olympus, Japan). The number of microglial cells in the three sections including the optic nerve, was counted under high magnification (×400) and averaged for each eye.

### Statistical analysis

The data were presented as mean ± standard deviation and analyzed using one-way analysis of variance (ANOVA) with SPSS software (version 16.0). For multiple comparisons, the Bonferroni test was employed. Statistical significance was set at *p* < 0.05.

## Results

### Effects of H_2_ on retinal function in *rd1* mice

**FIGURE 2:**
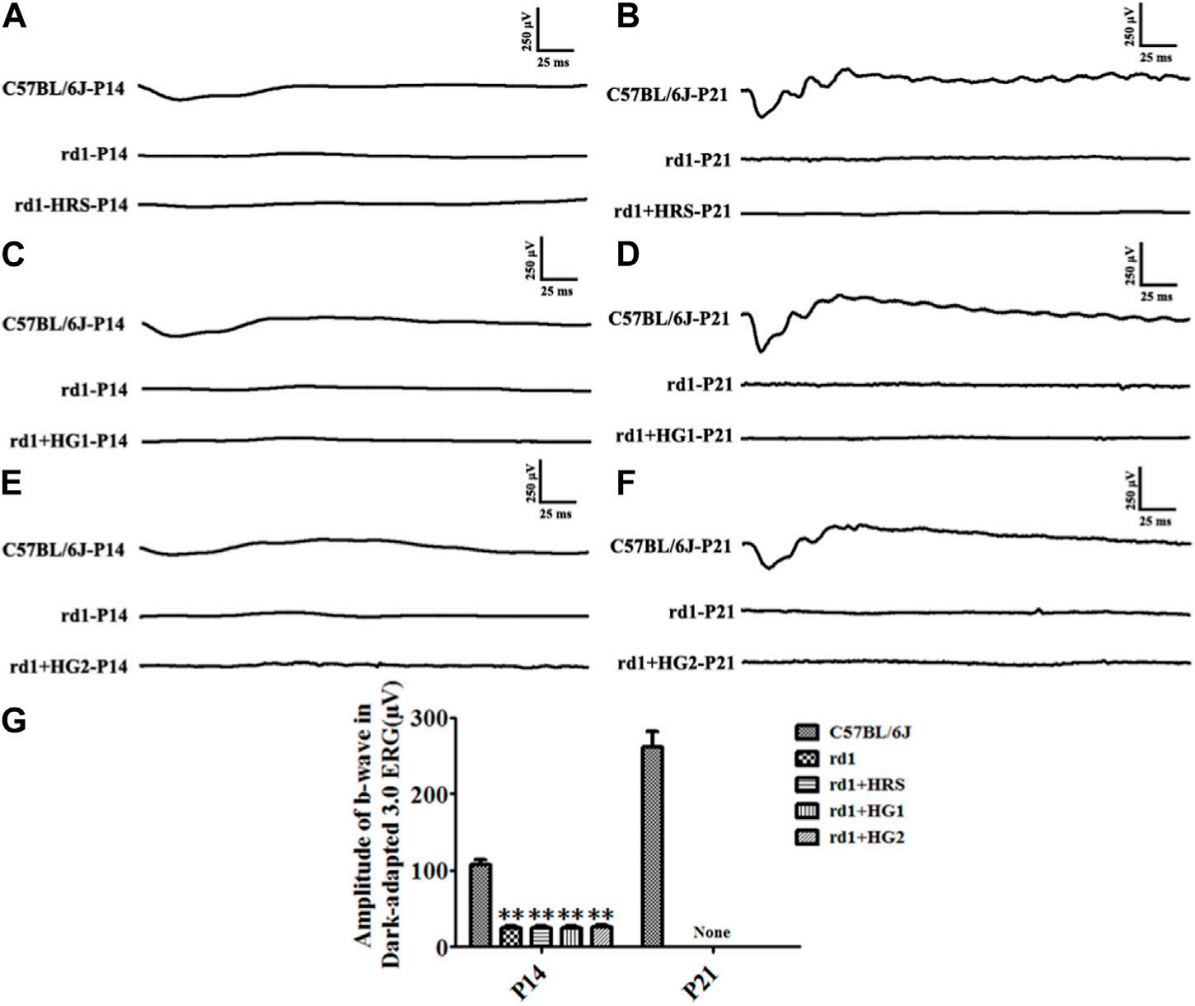
Effect of hydrogen intervention on the retinal function of *rd1* mice. Typical ERG waveforms of *rd1* mice at P14 and P21 after of intraperitoneal administration hydrogen-rich saline (HRS) **(A, B)**, after hydrogen gas inhalation for 4 h per day (HG1) **(C, D)**, or after hydrogen gas inhalation for 24 h per day (HG2) **(E, F)**. **(G)**: The statistical analysis of the b-wave amplitude in the Dark-adapted 3.0 ERG responses of *rd1* mice after different forms of hydrogen intervention. HRS: Hydrogen-rich saline intervention; HG1: Hydrogen gas inhalation for 4 h per day; HG2: Hydrogen gas inhalation for 24 h per day; **: *p <* 0.01 vs. C57BL/6J mice; None: No detection. Data were representative of 6 independent experiments.

The *rd1* mice from the control group had no discernible ERG waveforms at P14 and P21 compared to normal C57BL/6J mice. *rd1* mice from the HRS, HG1, and HG2 groups showed no obvious waveforms at P14 and P21. The b-wave amplitudes of the dark-adapted 3.0 ERG response in *rd1* mice in the control, HRS, HG1, and HG2 groups were significantly decreased compared to those in normal mice at both P14 and P21 (*p <* 0.01). However, there were no differences among *rd1* mice in the control, HRS, HG1, and HG2 groups (*p >* 0.05) (Figure 2).

### Effect of H_2_ on retinal morphology in *rd1* mice

**FIGURE 3:**
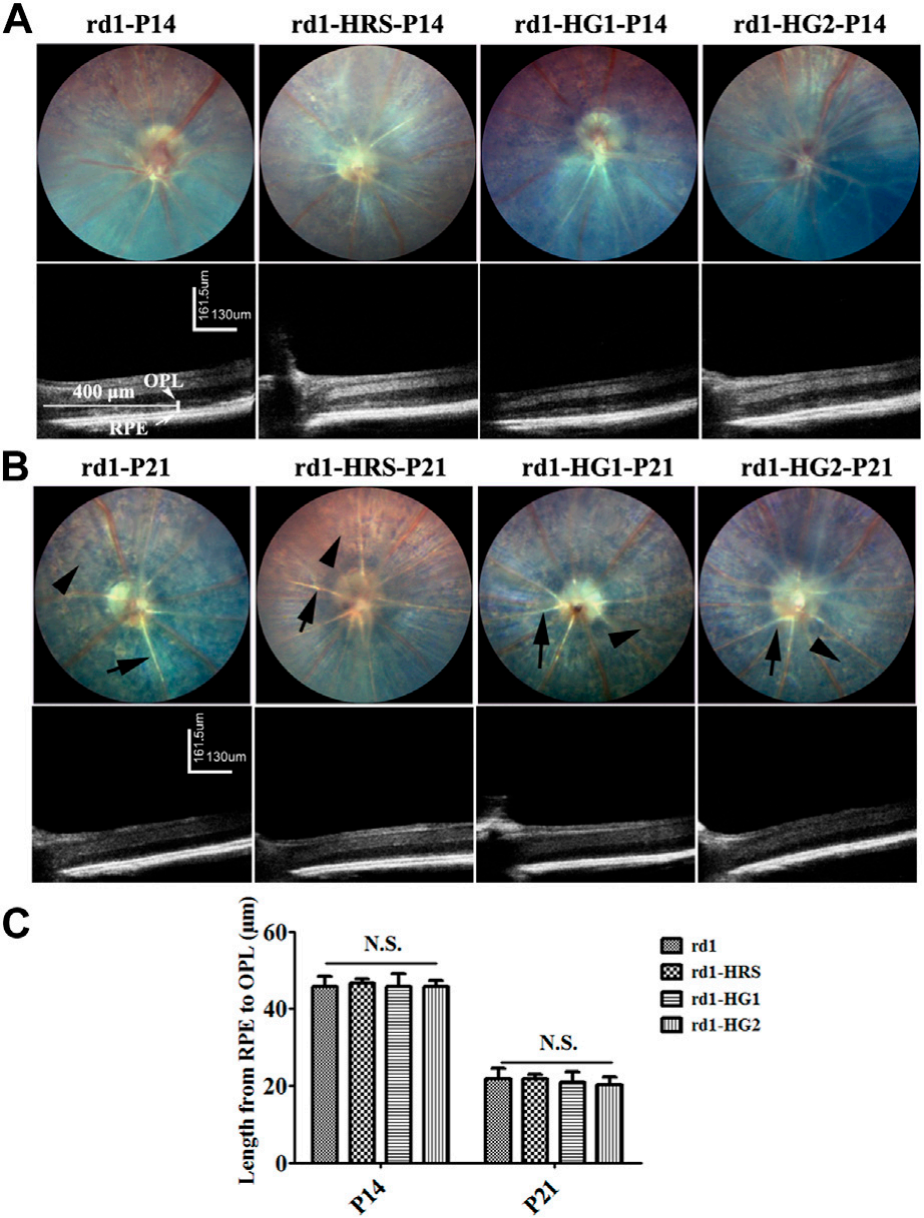
Effect of hydrogen intervention on fundus and the alive retinal morphology in the *rd1* mice. The typical images of ocular fundus and OCT images of *rd1* mice with hydrogen intervention at P14 **(A)** and P21 **(B)** after birth and the statistical analysis of the distance from RPE to OPL **(C)**. HRS: Hydrogen-rich saline; HG1: Hydrogen gas inhalation for 4 h per day; HG2: Hydrogen gas inhalation for 24 h per day; OCT: optical coherence tomography; RPE: retinal pigment epithelium; OPL: outer plexiform layer. N.S.: No significant differences. Data were representative of 6 independent experiments.

Typical RP phenotypes of *rd1* mice from the control group were observed under fundus especially at P21, including depigmentation, retinal vascular atrophy, and sclerosis. The ONL of the mice showed a high-density shadow on the OCT image at P14. Gradually, the ONL almost completely disappeared at P21. *rd1* mice from the HRS, HG1, HG2 group the ONL of *rd1* mice still showed degenerative retinal morphology with signs of retinal vascular sclerosis and pigmentation disorders. The ONL under OCT showed a high-density shadow at P14 in *rd1* mice in the HRS, HG1, and HG2 groups. There were no significant differences in the distance between the RPE and OPL among *rd1* mice from the control, HRS, HG1, and HG2 groups (*p >* 0.05) (Figure 3).

**FIGURE 4:**
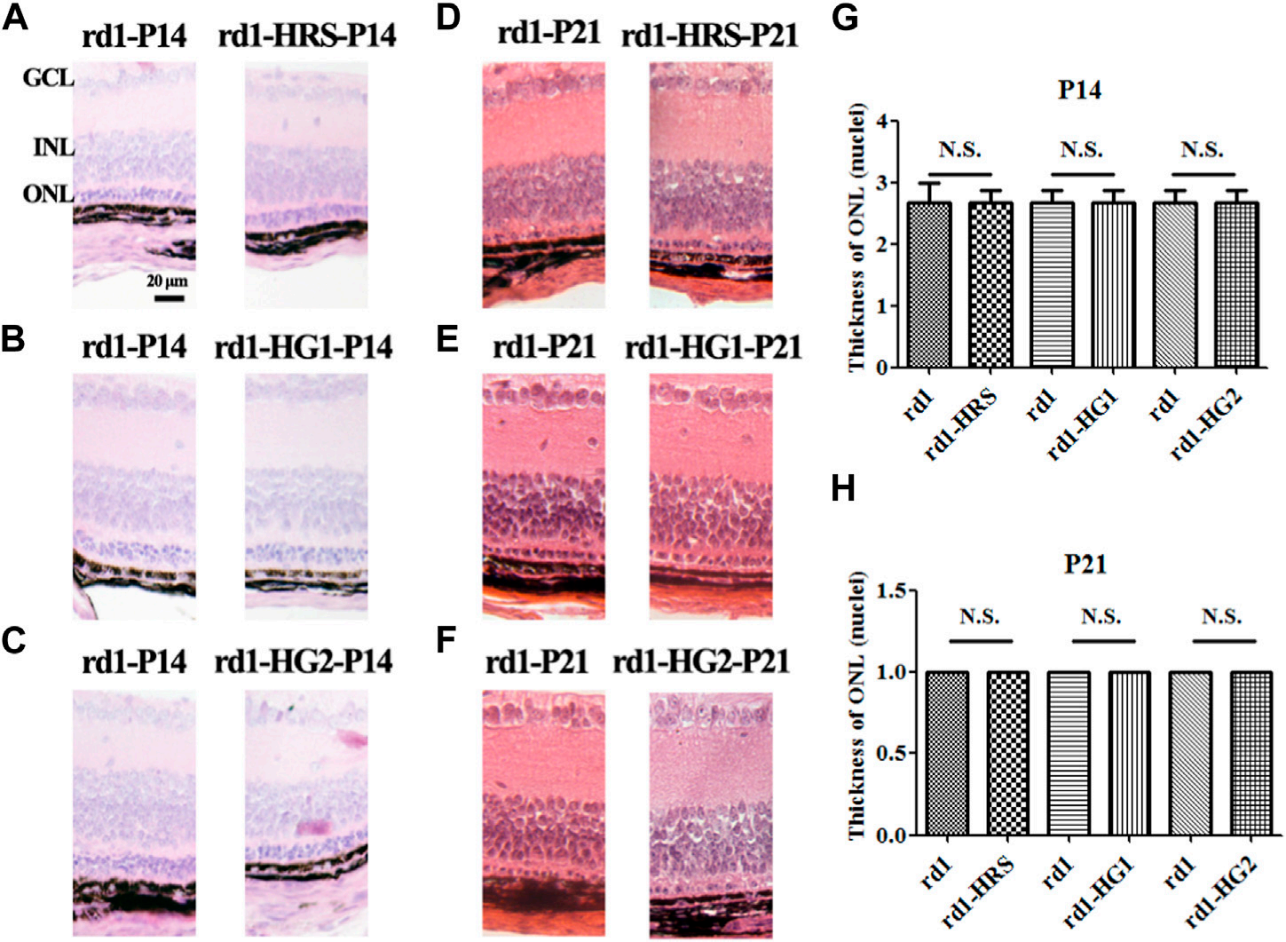
Effect of hydrogen intervention on retinal morphology in the *rd1* mice. Typical retinal sections with HE staining in *rd1* mice at P14 and P21 after intraperitoneal administration of hydrogen-rich saline (HRS) **(A, D)**, after hydrogen gas inhalation for 4 h per day (HG1) **(B, E)**, or after hydrogen gas inhalation for 24 h per day (HG2) **(C, F)**. **(G, H)**:The statistical analysis of ONL thickness (nuclei) of retinas from *rd1* mice after different forms of hydrogen intervention at P14 and P21. HRS: Hydrogen-rich saline; HG1: Hydrogen gas inhalation for 4 h per day; HG2: Hydrogen gas inhalation for 24 h per day; HE: hematoxylin and eosin; ONL: outer nuclear layer. N.S.: No significant differences. Data were representative of 6 independent experiments.

HE staining of paraffin sections of retinal pathological tissue showed that only three to four layers of ONL cells were left in the *rd1* mice of the control group at P14. Only one layer of ONL cells remained at P21 in the mice. Similarly, three to four layers of ONL cells remained at P14, and only one layer remained at P21 in *rd1* mice from the HRS, HG1, and HG2 groups. There were no significant differences in the number of ONL cell layers among the *rd1* mice in the control, HRS, HG1, and HG2 groups (*p >* 0.05) (Figure 4).

### Effect of H_2_ on the activation of microglia cells in *rd1* mice retinas

**FIGURE 5:**
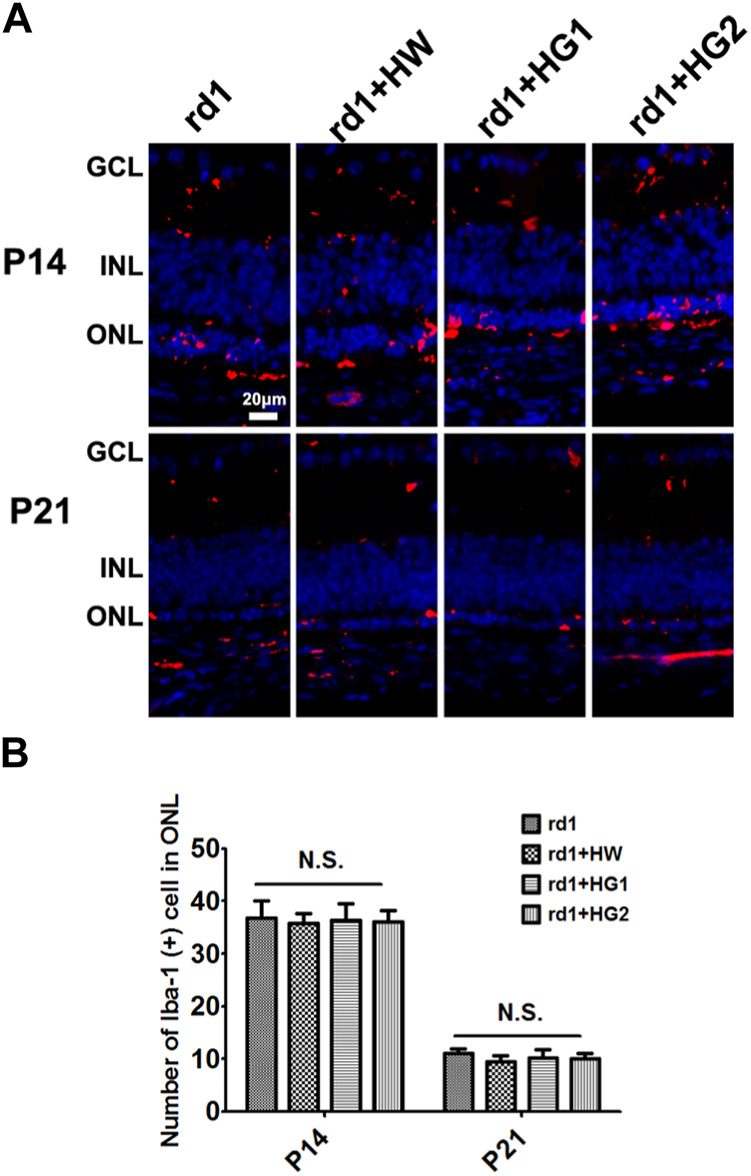
Effect of hydrogen intervention on microglia cells activation in the retinas of *rd1* mice. Typical images **(A)** of Iba1 immunostaining of microglia cells in the retina of *rd1* mice with hydrogen intervention and the statistical analysis of the Iba1-positive cells number in the retinas **(B)** at P14 and P21after intraperitoneal administration of hydrogen-rich saline (HRS), hydrogen gas inhalation for 4 h per day (HG1), or after hydrogen gas inhalation for 24 h per day (HG2). HRS: Hydrogen-rich saline; HG1: Hydrogen gas inhalation for 4 h per day; HG2: Hydrogen gas inhalation for 24 h per day: N.S.: No significant differences. Data were representative of 6 independent experiments.

The results of immunofluorescence staining of the retinal microglia cells by the specific marker Iba1 showed that the number of microglia cells in the retina of *rd1* mice from the control group at P21 was lower than that at P14 (*p <* 0.05). The number of Iba1-positive cells in the retinas of *rd1* mice at P14 and P21 was similar to that in mice from the HRS, HG1, and HG2 groups. No significant differences were found in the number of Iba1-positive cells in the retina among *rd1* mice from the control, HRS, HG1, and HG2 groups (*p >* 0.05) (Figure 5).

## Discussion

In this study, *rd1* mice, an inherited RP model, were treated with different forms of H_2_ for various durations. The hydrogen gas applied in our study was produced through a hydrogen-oxygen atomizer via electrolysis of water, which led to a gas mixture of hydrogen-oxygen (67% H_2_ and 33% O_2_) (Yan et al., 2018). The concentration of oxygen was then diluted to be consistent with the normal atmosphere, and the final concentration of hydrogen gas at this time was 44%. In view of the available literature, no clear reports on the dose effect of H_2_-induced biological effects have been published. Therefore, only a single hydrogen concentration was used in this study.

The pathogenic factor in *rd1* mice is a nonsense mutation in the *Pde6b* gene, resulting in the loss of the PDE6B protein (Nishiguchi et al., 2015). Defective PDE6B protein expression in cells leads to reduced cGMP hydrolysis and increased cGMP levels, causing intracellular calcium overload and subsequent photoreceptors death (Yan W. et al., 2017). Apoptosis has been shown to be responsible for the photoreceptors death in the retina of *rd1* mice (Wang et al., 2019). ROS are known to play an important role in photoreceptor apoptosis, which can damage the cell membrane and release inner components, such as Cytochrome C, cathepsin, and other hydrolases (Pan et al., 2020; Kanan et al., 2022). Other studies have reported that increased glycolytic enzyme activity in *rd1* mice leads to increased ROS production and oxidative stress (Acosta et al., 2005). Some antioxidant substances were argued to be able to delay the process of *rd1* mice to a certain extent by reducing oxidative stress, including lutein, zeaxanthin, and α-lipoic acid (Sahu et al., 2021). In addition, *in-vitro* culture of photoreceptor cells revealed that antioxidants rescued the cells from apoptosis (Chen et al., 2017). The retinal function of *rd1* mice in our study was detected via ERG recording, with the retinal morphology observed using live OCT, fundus photography, and retinal histological sections. However, our data showed that H_2_, which also has antioxidant properties, was unable to alleviate retinal degeneration phenotypes or photoreceptor apoptosis in *rd1* mice. In addition, photoreceptor apoptosis of the retina in rd1 mice was monitored using SIRT1 protein electrophoresis. SIRT1, which could exist effective biological effects through antioxidant and antiapoptosis mechanisms (Zhang et al., 2022b; Li et al., 2022), has been shown to be downregulated in various animal models of retinal degeneration (Yan et al., 2020). We previously found that SIRT1 was also decreased in the *rd1* mice compared to normal mice. Besides, H_2_ was showed to upregulate the SIRT1 protein level and reduced photoreceptors damage in a MNU-induced RP rat model (Yan et al., 2017a). However, the result of our present study found that H_2_ did not significantly upregulate the level of SIRT1 protein in the retina of *rd1* mice (data shown in Supplementary Figure S2). Additionally, no significant effect on PDE6B protein expression, which is the underlying pathogenetic mechasism of rd1 mice, was observed by the H_2_ intervention (data shown in Supplementary Figure S2).

Microglia activation, a sign of neuroinflammation, has been reported in the retinas of *rd1* mice (Zhou et al., 2017). A medication that inhibits microglial activation has been demonstrated to attenuate photoreceptor apoptosis (Wang et al., 2017). H_2_ was previously argued to reduce microglial activation in other disease models (Zhao et al., 2020), whereas the results of our study suggest that it did not significantly interfere with the numbers of microglial cells in the retina of *rd1* mice. However, changes in microglial cell number may not necessarily reflect changes in microglial activation state, which requires further investigation. Indeed, in another type of RP mouse model, the *rd6* mouse, researchers investigated the effects of hydrogen water intervention. Their results revealed that hydrogen water could improve visual function and morphology, but it was unable to alter the quantity of retinal microglial cells (Igarashi et al., 2022).

In view of the above results, one possible reason for the failure of H_2_-induced relief in *rd1* mice may be the rapid apoptosis of photoreceptors. Apoptosis of photoreceptors began from the birth of *rd1* mice, and the photoreceptor cells in *rd1* mice almost disappeared within 21 days. H_2_ is a relatively mild antioxidant that selectively neutralizes the strong ROS such as, ·OH and ONOO– in the cellular structure (Ohsawa et al., 2007; Tian et al., 2021). H_2_ intervention may not be sufficient to produce the expected beneficial effects. Another inherited RP model, *rd10* mice (Olivares-Gonzalez et al., 2020), with a later onset and slow progression of photoreceptor apoptosis, might provide a longer window period for medication intervention.

Another possible reason for the failure of H_2_-induced relief might lie in the special mechanism of photoreceptor apoptosis in the inherited RP model of *rd1* mice compared to the induced RP models (Wei et al., 2021). The primary factors for photoreceptor apoptosis in *rd1* mice caused by nonsense mutations in *Pde6b* could not be altered by H_2_. Some medications that had a beneficial effect on induced retinal degeneration models failed to rescue retinal degeneration in inherited genetic models. For example, systemic administration of recombinant human erythropoietin (EPO) or an increased EPO level through hypoxia induction protected photoreceptor cells in a mouse model of light-induced retinal degeneration (Grimm et al., 2004). However, increased EPO levels via intraperitoneal injection of human recombinant erythropoietin or subretinal injection of the adeno-associated virus (AAV)-Epo gene did not affect photoreceptor apoptosis in *rd1* mice. Transgenic overexpression of the EPO gene alleviated photoreceptor apoptosis in a mouse model of light-damaged retina, which reduced the degree of retinal degeneration (Grimm et al., 2004). Moreover, elevated EPO could achieve a relief effect on retinal degeneration in *rds* mice, another retinal degeneration mouse model. However, no obvious intervention effects were observed in the *rd1*0 mice (Rex et al., 2004). The above evidence suggests that specific mechanisms of photoreceptor apoptosis might exist in different retinal degeneration models, which have different sensitivities to medical interventions.

There were some limitations of our study. One of the limitations of our study was that we chose to administer hydrogen-rich saline via intraperitoneal injection to intervene the retinal degeneration of rd1 mice 7 days after birth. This decision was based on the relative developmental size of the mice at 7 days after birth, making the intraperitoneal injection procedure relatively easier. In our future studies, we would attempt to administer the intervention via intraperitoneal injection at an earlier time. Another limitation was that we did not directly measure the levels of reactive oxygen species in the retinas of the mice, nor did we assess the content of oxidative stress-related enzymes. Instead, we only examined the expression levels of the oxidative stress-regulating protein SIRT1 (data shown in Supplementary Figure S2). This aspect requires further improvement in our subsequent research.

In conclusion, our study found that H_2_ had no obvious effect on retinal degeneration in an inherited RP model of *rd1* mice. Photoreceptor apoptosis in different types of RP models may require different medical interventions, which reflects the complexity of pathogenesis and requires further exploration.

## Data Availability

The raw data supporting the conclusion of this article will be made available by the authors, without undue reservation.

## References

[B1] Acosta M. L. Fletcher E. L. Azizoglu S. Foster L. E. Farber D. B. Kalloniatis M. (2005). Early markers of retinal degeneration in rd/rd mice. Mol. Vis. 11, 717–728. 16163270

[B2] Alwazeer D. Liu F. F. Wu X. Y. LeBaron T. W. (2021). Combating oxidative stress and inflammation in COVID-19 by molecular hydrogen therapy: mechanisms and perspectives. Oxid. Med. Cell. Longev. 2021, 5513868. 10.1155/2021/5513868 34646423 PMC8505069

[B3] Chen T. Tao Y. Yan W. Yang G. Chen X. Cao R. (2016). Protective effects of hydrogen-rich saline against N-methyl-N-nitrosourea-induced photoreceptor degeneration. Exp. EYE Res. 148, 65–73. 10.1016/j.exer.2016.05.017 27215478

[B4] Chen W. J. Wu C. Xu Z. Kuse Y. Hara H. Duh E. J. (2017). Nrf2 protects photoreceptor cells from photo-oxidative stress induced by blue light. Exp. EYE Res. 154, 151–158. 10.1016/j.exer.2016.12.001 27923559 PMC6054877

[B5] Daiger S. P. Sullivan L. S. Bowne S. J. (2013). Genes and mutations causing retinitis pigmentosa. Clin. Genet. 84 (2), 132–141. 10.1111/cge.12203 23701314 PMC3856531

[B6] Dvoriantchikova G. Lypka K. R. Ivanov D. (2022). The potential role of epigenetic mechanisms in the development of retinitis pigmentosa and related photoreceptor dystrophies. Front. Genet. 13, 827274. 10.3389/fgene.2022.827274 35360866 PMC8961674

[B7] Grimm C. Wenzel A. Stanescu D. Samardzija M. Hotop S. Groszer M. (2004). Constitutive overexpression of human erythropoietin protects the mouse retina against induced but not inherited retinal degeneration. J. Neurosci. 24 (25), 5651–5658. 10.1523/JNEUROSCI.1288-04.2004 15215287 PMC2929919

[B8] Igarashi T. Ohsawa I. Kobayashi M. Miyazaki K. Igarashi T. Kameya S., (2022). Drinking hydrogen water improves photoreceptor structure and function in retinal degeneration 6 mice. Sci. Rep. 12 (1), 13610. 10.1038/s41598-022-17903-8 35948585 PMC9365798

[B9] Kanan Y. Hackett S. F. Taneja K. Khan M. Campochiaro P. A. (2022). Oxidative stress-induced alterations in retinal glucose metabolism in Retinitis Pigmentosa. Free Radic. Biol. Med. 181, 143–153. 10.1016/j.freeradbiomed.2022.01.032 35134532 PMC8891093

[B10] Leclercq B. Hicks D. Laurent V. (2021). Photoperiod integration in C3H rd1 mice. J. PINEAL Res. 71 (2), 12711. 10.1111/jpi.12711 33326640

[B11] Li Q. Wu J. Huang J. Hu R. You H. Liu L. (2022). Paeoniflorin ameliorates skeletal muscle atrophy in chronic kidney disease *via* AMPK/SIRT1/PGC-1α-Mediated oxidative stress and mitochondrial dysfunction. Front. Pharmacol. 13, 859723. 10.3389/fphar.2022.859723 35370668 PMC8964350

[B12] Liu W. Liu S. Li P. Yao K. (2022). Retinitis pigmentosa: progress in molecular pathology and biotherapeutical strategies. Int. J. Mol. Sci. 23 (9), 4883. 10.3390/ijms23094883 35563274 PMC9101511

[B13] McCulloch D. L. Marmor M. F. Brigell M. G. Hamilton R. Holder G. E. Tzekov R. (2015). ISCEV Standard for full-field clinical electroretinography (2015 update). Doc. Ophthalmol. 130 (1), 1–12. 10.1007/s10633-014-9473-7 25502644

[B14] Nishiguchi K. M. Carvalho L. S. Rizzi M. Powell K. Holthaus S. M. Azam S. A. (2015). Gene therapy restores vision in rd1 mice after removal of a confounding mutation in Gpr179. Nat. Commun. 6, 6006. 10.1038/ncomms7006 25613321 PMC4354202

[B15] Nogueira J. E. Branco L. (2021). Recent advances in molecular hydrogen research reducing exercise-induced oxidative stress and inflammation. Curr. Pharm. Des. 27 (5), 731–736. 10.2174/1381612826666201113100245 33185152

[B16] Ohsawa I. Ishikawa M. Takahashi K. Watanabe M. Nishimaki K. Yamagata K. (2007). Hydrogen acts as a therapeutic antioxidant by selectively reducing cytotoxic oxygen radicals. Nat. Med. 13 (6), 688–694. 10.1038/nm1577 17486089

[B17] Olivares-Gonzalez L. Velasco S. Millan J. M. Rodrigo R. (2020). Intravitreal administration of adalimumab delays retinal degeneration in rd10 mice. FASEB J. 34 (10), 13839–13861. 10.1096/fj.202000044RR 32816354

[B18] Pan Y. R. Song J. Y. Fan B. Wang Y. Che L. Zhang S. M. (2020). mTOR may interact with PARP-1 to regulate visible light-induced parthanatos in photoreceptors. Cell. Commun. SIGNAL 18 (1), 27. 10.1186/s12964-019-0498-0 32066462 PMC7025415

[B19] Rex T. S. Allocca M. Domenici L. Surace E. M. Maguire A. M. Lyubarsky A. (2004). Systemic but not intraocular Epo gene transfer protects the retina from light-and genetic-induced degeneration. Mol. Ther. 10 (5), 855–861. 10.1016/j.ymthe.2004.07.027 15509503

[B20] Sahu B. Leon L. M. Zhang W. Puranik N. Periasamy R. Khanna H. (2021). Oxidative stress resistance 1 gene therapy retards neurodegeneration in the rd1 mutant mouse model of retinopathy. Invest. Ophthalmol. Vis. Sci. 62 (12), 8. 10.1167/iovs.62.12.8 PMC8434758 34505865

[B21] Tian Y. Zhang Y. Wang Y. Chen Y. Fan W. Zhou J. (2021). Hydrogen, a novel therapeutic molecule, regulates oxidative stress, inflammation, and apoptosis. Front. Physiol. 12, 789507. 10.3389/fphys.2021.789507 34987419 PMC8721893

[B22] Vingolo E. M. Casillo L. Contento L. Toja F. Florido A. (2022). Retinitis pigmentosa (RP): the role of oxidative stress in the degenerative process progression. Biomedicines 10 (3), 582. 10.3390/biomedicines10030582 35327384 PMC8945005

[B23] Wang L. Shi K. P. Li H. Huang H. Wu W. B. Cai C. S. (2019). Activation of the TRAAK two-pore domain potassium channels in rd1 mice protects photoreceptor cells from apoptosis. Int. J. Ophthalmol. 12 (8), 1243–1249. 10.18240/ijo.2019.08.03 31456913 PMC6694065

[B24] Wang Y. Yin Z. Gao L. Sun D. Hu X. Xue L. (2017). Curcumin delays retinal degeneration by regulating microglia activation in the retina of rd1 mice. Cell. PHYSIOL. Biochem. 44 (2), 479–493. 10.1159/000485085 29145208

[B25] Wei C. Li Y. Feng X. Hu Z. Paquet-Durand F. Jiao K. (2021). RNA biological characteristics at the peak of cell death in different hereditary retinal degeneration mutants. Front. Genet. 12, 728791. 10.3389/fgene.2021.728791 34777465 PMC8586524

[B26] Yan W. Chen T. Long P. Zhang Z. Liu Q. Wang X. (2018). Effects of post-treatment hydrogen gas inhalation on uveitis induced by endotoxin in rats. Med. Sci. Monit. 24, 3840–3847. 10.12659/MSM.907269 29875353 PMC6020745

[B27] Yan W. Long P. Wei D. Yan W. Zheng X. Chen G. (2020). Protection of retinal function and morphology in MNU-induced retinitis pigmentosa rats by ALDH2: an *in-vivo* study. BMC Ophthalmol. 20 (1), 55. 10.1186/s12886-020-1330-8 32070320 PMC7027227

[B28] Yan W. Yao L. Liu W. Sun K. Zhang Z. Zhang L. (2017b). A kind of rd1 mouse in C57BL/6J mice from crossing with a mutated Kunming mouse. GENE 607, 9–15. 10.1016/j.gene.2017.01.006 28077313

[B29] Yan W. M. Chen T. Wang X. C. Qi L. S. Zhao G. H. Yang G. Q. (2017a). The reason for the amelioration of N-methyl-N-nitrosourea-induced retinitis pigmentosa in rats by hydrogen-rich saline. Int. J. Ophthalmol. 10 (10), 1495–1503. 10.18240/ijo.2017.10.03 29062766 PMC5638968

[B30] Yan W. M. Long P. Chen M. Z. Wei D. Y. Wang J. C. Zhang Z. M. (2021). Retinal neovascularization induced by mutant Vldlr gene inhibited in an inherited retinitis pigmentosa mouse model: an *in-vivo* study. Int. J. Ophthalmol. 14 (7), 990–997. 10.18240/ijo.2021.07.05 34282382 PMC8243196

[B31] Yan W. M. Zhang L. Chen T. Zhao G. H. Long P. An J. (2017c). Effects of hydrogen-rich saline on endotoxin-induced uveitis. Med. Gas. Res. 7 (1), 9–18. 10.4103/2045-9912.202905 28480027 PMC5402351

[B32] Zhang Y. Li T. Pan M. Wang W. Huang W. Yuan Y. (2022b). SIRT1 prevents cigarette smoking-induced lung fibroblasts activation by regulating mitochondrial oxidative stress and lipid metabolism. J. Transl. Med. 20 (1), 222. 10.1186/s12967-022-03408-5 35568871 PMC9107262

[B33] Zhang Y. Zhang J. Fu Z. (2022a). Molecular hydrogen is a potential protective agent in the management of acute lung injury. Mol. Med. 28 (1), 27. 10.1186/s10020-022-00455-y 35240982 PMC8892414

[B34] Zhao Q. H. Xie F. Guo D. Z. Ju F. D. He J. Yao T. T. (2020). Hydrogen inhalation inhibits microglia activation and neuroinflammation in a rat model of traumatic brain injury. Brain Res. 1748, 147053. 10.1016/j.brainres.2020.147053 32814064

[B35] Zhou T. Huang Z. Sun X. Zhu X. Zhou L. Li M. (2017). Microglia polarization with M1/M2 phenotype changes in rd1 mouse model of retinal degeneration. Front. Neuroanat. 11, 77. 10.3389/fnana.2017.00077 28928639 PMC5591873

