# Supplementary material for: Effect of molecular hydrogen, a novelly-established antioxidant, on the retinal degeneration of hereditary retinitis pigmentosa: an in-vivo study
Source: Front Pharmacol. 2024 Apr 3;14:1294315. doi: 10.3389/fphar.2023.1294315 (PMC11025393; doi:10.3389/fphar.2023.1294315)
Supplement: Supplementary file 1 [file DataSheet1.docx]

**Supplements:**


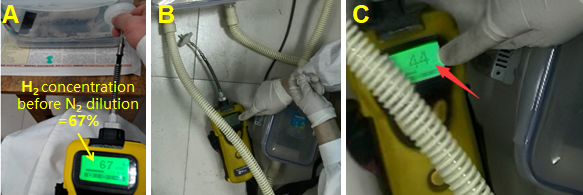


**Supplement Figure 1.** **The initial undiluted concentration of hydrogen gas in the cage and the concentration after dilution.** A: the H_2_ value detected before the dilution with nitrogen gas was 67% (the yellow arrow) in the cage. B: the photographs taken during the study, which displayed the hydrogen gas concentration after dilution. C: a concentration of approximately 44% (the red arrow) of hydrogen gas after enlargement of Part B.

**Supplement Table 1. The concentration of the H_2_ values monitored in the animals' cages throughout the research**

For HG1:

| **Times** | 08：00 am | 9：00 am | 11:00 am | 12:00 am |
| --- | --- | --- | --- | --- |
| **H_2_ values** | 44% | 44% | 44% | 44% |

For HG2:

| **Times** | 08：00 am | 12：00 am | 4:00 pm | 8:00 pm | 12 pm | 4:00 am | 8:00 am |
| --- | --- | --- | --- | --- | --- | --- | --- |
| **H_2_ values** | 44% | 44% | 44% | 44% | 44% | 44% | 44% |

**Note:** HG1: Hydrogen gas inhalation for 4 h per day; HG2: Hydrogen gas inhalation for 24 h per day.

**Supplement 3: Effect of H**_2_ **on the SIRT1 and PDE6B proteins expression in *rd1* mice retinas**

Western blot detection showed that PDE6B protein was not expressed in the retina of all the *rd1* mice from the control, the HRS, the HG1, and the HG_2_ groups. SIRT1 protein was detected in the retinas of the *rd1* mice. However, there were no statistical differences in the expression level of SIRT1 protein in the retina of among the *rd1* mice from the control, the HRS, the HG1, and the HG_2_ groups (*P >* 0.05). (**Supplement Figure 2**)


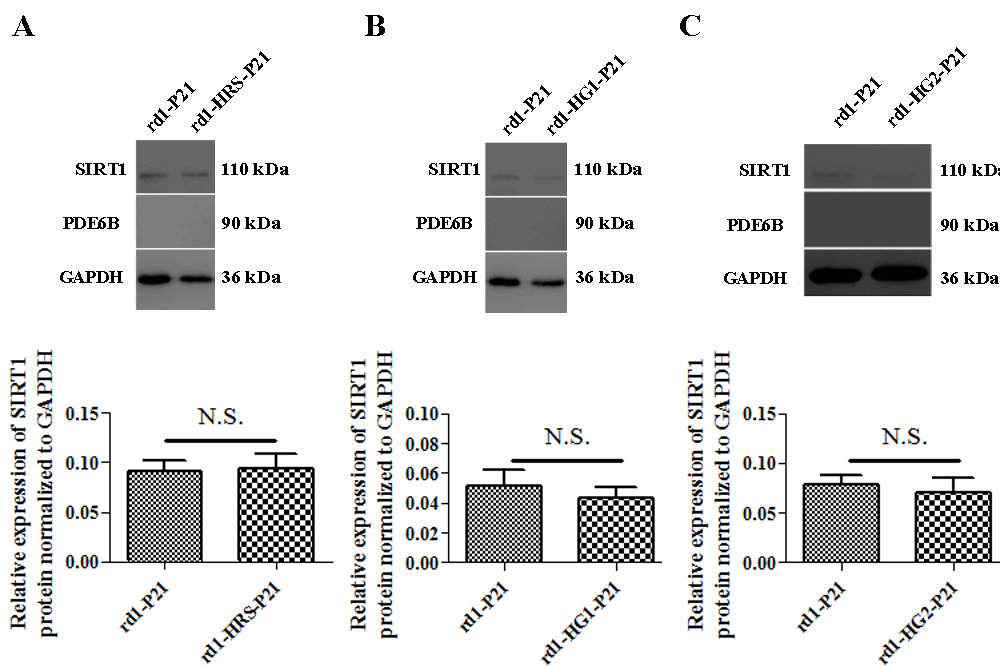


**Supplement Figure 2. Effect of hydrogen intervention on the expression of PDE6B and SIRT1 in the retina of *rd1* mice.** Typical Western blot bands and quantitative analysis of PDE6B and SRIT1 protein in the *rd*1 mice’s retinas after intraperitoneal administration of hydrogen-rich saline (HRS) (A), hydrogen gas inhalation for 4 h per day (HG1) (B), or after hydrogen gas inhalation for 24 h per day (HG2) (C). There was no expression of PDE6B in the retina of *rd1* mice after hydrogen intervention. The relative expression of SIRT1 in *rd1* mice with hydrogen intervention was not significantly different from that without intervention. HRS: Hydrogen-rich saline; HG1: Hydrogen gas inhalation for 4 h per day; HG2: Hydrogen

gas inhalation for 24 h per day; N.S.: No significant differences.
